# Statewide implementation of a quality improvement initiative for reproductive, maternal, newborn and child health and nutritionin Bihar, India

**DOI:** 10.7189/jogh.10.021008

**Published:** 2020-12

**Authors:** Andreea A Creanga, Sridhar Srikantiah, Tanmay Mahapatra, Aritra Das, Sunil Sonthalia, Prabir Ranjan Moharana, Aboli Gore, Sanjiv Daulatrao, Rohini Durbha, Sunil Kaul, Christine Galavotti, Anne Laterra, Kevin T Pepper, Gary L Darmstadt, Hemant Shah

**Affiliations:** 1 Department of International Health, Johns Hopkins Bloomberg School of Public Health, Baltimore, Maryland, USA; 2 Department of Gynecology and Obstetrics, Johns Hopkins School of Medicine, Baltimore, Maryland, USA; 3 CARE India Solutions for Sustainable Development, Patna, Bihar, India; 4 CARE USA, Atlanta, Georgia, USA; 5 Department of Pediatrics, Stanford University School of Medicine, Stanford, California, USA

## Abstract

**Background:**

CARE India designed and implemented a comprehensive, statewide quality improvement (QI) initiative to improve reproductive, maternal, newborn, and child health and nutrition (RMNCHN) services in public facilities in Bihar. We provide a description of this initiative and its key results during 2014-2017.

**Methods:**

We reviewed program documents to identify QI strategies employed and ascertain their coverage. We analysed data from: a) two public facility assessments to ascertain the availability of essential equipment and supplies and the distribution of human resources by facility level; b) a four-phase provider mentoring and training intervention covering 319 facilities to examine changes in emergency obstetric and newborn care (EmONC) practices; and c) four state-representative household surveys to explore changes in selected RMNCHN service utilisation by health sector. Associations of interest were ascertained using χ^2^ tests.

**Results:**

Thirty-eight District Quality Assurance Committees and QI teams in 98% of facilities were formed to develop an implementation plan for the QI initiative and oversee its execution. QI strategies were to strengthen facilities’ infrastructure; build the state’s contracting, procurement, and inventory management capacities; rationalise human resources; improve providers’ skills; and modernise data systems. Implementation led to facility infrastructure upgrades, improved clinical staff distribution, and higher availability of equipment and supplies over time, especially in higher-level facilities. Following the mentoring and training intervention in facilities offering both basic and comprehensive EmONC, performance of key practices (eg, adequate administration of uterotonics <1 minute after birth, initiation of skin-to-skin care <5 minutes after birth) improved significantly (*P* < 0.05). CARE India collected program data and assisted with modernising data systems for tracking human resources, supplies, and program progress statewide. Of women seeking antenatal care, the proportion obtaining key screenings (eg, weight, blood pressure measurements) in public facilities increased over time (*P* < 0.05). A 6-percentage point decline in home deliveries during 2016-2017 was accompanied by a higher increase of deliveries in public than private facilities (5- vs 1-percentage point; *P* < 0.05).

**Conclusion:**

Substantial advances were made in improving RMNCHN service quality in Bihar. Continued improvement building on the established QI platform is expected and should be guided by data from now functional data systems.

The health system in Bihar, one of India’s poorest and most densely populated states, has been facing systemic deficiencies for decades [1,2]. As described earlier in this series [1], CARE India, a leading global humanitarian organisation, and other non-governmental partners along with the Government of Bihar (GoB) embarked on the *Ananya* program in 2010. At that time, the delivery of health services in public facilities in Bihar was hindered by the fragile and insufficient infrastructure, severe shortage of skilled health providers, low health provider motivation, and lack of essential supplies and equipment [1]. It was widely recognised that the quality of reproductive, maternal, newborn, and child health and nutrition (RMNCHN) services offered in public facilities throughout the state were poor, especially in rural areas where 90% of the population live [3-8]. Yet, the disconnect between extremely low levels of coverage and quality of emergency obstetric and newborn care (EmONC) and available state-level estimates of maternal mortality (305 deaths per 100 000 live births) and neonatal mortality (35 deaths per 1000 live births) reported for 2010-2011 was striking [9]. Recognising that these indicators were likely underestimated and not reflecting the level of deficiencies in RMNCHN service delivery, beginning in 2011, CARE India, in partnership with the GoB, designed a comprehensive quality improvement (QI) initiative focused specifically on improving the quality of RMNCHN services in public facilities in Bihar to the point where they could contribute to effectively and consistently reducing mortality rates [8]. This complemented similarly directed strategies to improve outreach by frontline health workers and address service coverage and encourage behavior changes at the family level [1]. The QI initiative was initially launched in eight districts and scaled to all 38 districts within the state with support from the Bihar Technical Support Program (BTSP) in 2014 [1,8].

While QI initiatives in Bihar were implemented with guidance from CARE India in eight districts since 2011, they benefitted from a favourable national context that evolved over the following years. The RMNCHN + adolescent health (RMNCHN+A) strategy of the Government of India launched in 2013 included a comprehensive approach to implementing effective interventions with specific emphasis on a wide spectrum of health system components; National Quality Assurance guidelines for health facilities were also released the same year; and, in 2017, the Government of India launched the LaQshya guidelines, which reinforced the need for a QI approach for intrapartum care [10,11]. Because the standards demanded in the RMNCHN+A strategy were “aspirational goals” in Bihar in 2013, they drew attention from the GoB and other stakeholders, and created a momentum for broad health system and RMNCHN service delivery improvement. A separate GoB initiative called *Manav Vikas* Mission (translated Human Development Mission) also set ambitious health goals, including reductions in maternal and newborn mortality [8].

Early in the Sustainable Development Goals era, there is much interest to learn from the implementation of large QI initiatives in low- and middle-income countries – GoB’s QI initiative, catalyzed by CARE India, offers this opportunity. It is a particularly important opportunity given significant state-level changes in selected RMNCHN indicators [2-7,12,13], including a 4-fold increase in institutional delivery rates [8] reported between 2006 and 2011, before the QI work was initiated. This article provides a description of the QI initiative and its key results pertaining to RMNCHN service coverage and quality during 2014-2017, the time period when implementation was statewide.

## METHODS

We first reviewed CARE India’s program documents (eg, internal activity reports, donor reports, data briefs) to compile a comprehensive list of QI strategies employed in Bihar. Co-authors from CARE India were key to providing context and helping resolve discrepancies found in such large amounts of information. We extracted year-specific coverage information for relevant QI strategy indicators to document the coverage of the QI initiative during 2014-2017. To structure our manuscript, we organised the QI strategies under the following categories: management and coordination; facility infrastructure; contracting, procurement, and inventory management capacity; human resources; clinical skills; and health data systems.

We analysed data from multiple sources to assess and exemplify the key results of the QI strategies employed. These data sources are described in detail elsewhere in this series [13] and summarised in Supplemental Table 1in the **Online Supplementary Document**. Data from two Comprehensive Facility Assessments conducted in all public facilities in the state in 2015 (N = 532) and 2017 (N = 549) were used to assess and compare the availability of essential equipment and supplies as well as changes in the distribution of human resources by facility level (primary health centres (PHCs) vs higher-level facilities). Statistically significant differences between 2015 and 2017 were tested using Pearson χ^2^ tests.

Data from Direct Observations of Deliveries conducted before and after each of four phases of a mentoring and training intervention named *Apatkalin Matritva evam Navjat Tatparta* (AMANAT, translated Emergency Maternal and Neonatal Care Preparedness) implemented in 36 of 38 districts in the state were used to examine changes in four key obstetric and newborn care practices: blood pressure measurement before delivery, administration of uterotonics via proper dose and route within <1 minute of delivery, use of a sterile blade/scissors for cord cutting, and initiation of breastfeeding <1 hour after birth. Analyses were conducted stratified by intervention phase (I-IV), separately for facilities offering basic and comprehensive EmONC (BEmONC and CEmONC, respectively). Statistically significant baseline-endline differences were assessed using Pearson χ^2^ tests for each phase.

Finally, data from four state-representative Community-based Household Surveys conducted annually during 2014-2017 (total sample 62 685 women interviewed; ~ 15 671 women with a live child 0-2 months per survey round) were used to explore changes in service utilisation over time: a) antenatal care (ANC, ie, any check-up, 3+, and 4+ check-ups) and content (ie, receipt of blood test, receipt of urine test, blood pressure check, weight measurement, abdominal examination, ultrasound examination); b) place of delivery (ie, home vs facility) and use of ambulances to reach public facilities for delivery; c) caesarean delivery; and d) essential newborn care (ie, skin-to-skin practice, initiation of breastfeeding <1 hour after birth, newborn weighing, and dry cord care). Changes in service utilisation were assessed over time, overall and by health sector (public vs private facilities). Cuzick tests-for-trend were employed to ascertain whether the changes observed between 2014 and 2017 were statistically significant at *P* < 0.05. Given the complex survey design, we used Taylor’s linearisation method for variance estimation for all our indicators of interest.

All analyses were conducted using Stata version 14. The Institutional Committee for Ethics and Review of Research of the Indian Institute of Health Management Research in Jaipur, India approved data collection activities undertaken by CARE India during 2014-2015. The Ashirwad Ethics Committee at the Ashirwad Hospital and Research Center in Ulhasnagar, India did so for data collection activities in 2016 and subsequently. This study involved analysis of de-identified secondary data from CARE India obtained under a data use agreement and was deemed not human subjects research by the Institutional Review Board at the Johns Hopkins School of Public Health.

## RESULTS

### General design and implementation considerations

#### Approach

The overarching goal of the initiative was to improve the quality of RMNCHN clinical care and outcomes. The QI initiative was implemented in all public facilities ( ~ 550) in Bihar, from first-level delivery points represented by block-level PHCs to district hospitals. Selection of specific strategies and interventions to implement in health facilities had to account for the relatively high level of institutional deliveries (72% in 2014), driven by the *Janani Suraksha Yojana* (JSY) cash transfer scheme that increased institutional births throughout India [2,4,14]. Since most of these deliveries were taking place in public facilities, the decision was made for the program to emphasise strengthening EmONC services in these facilities. A multi-dimensional approach was chosen to concurrently and incrementally strengthen facilities’ infrastructure; improve the availability of essential equipment and supplies needed, which involved strengthening the state’s contracting, procurement, and inventory management capacity; ensure a more adequate distribution of available human resources; provide intensive, on-site clinical mentoring and training for nurses and doctors to improve their clinical skills and compliance with evidence-based practices; document basic care and QI processes as well as health outcomes; and use data for continuous planning, monitoring, supervision, and course-correction of each QI strategy. Learnings from an initial eight-district pilot phase were used to design the QI initiative statewide, allowing for adaptation based on local context and specific needs to overcome systemic barriers to quality RMNCHN service delivery. Improvement methods in the form of Plan-Do-Study-Act cycles were attempted throughout 2014-2017 with assistance from CARE India as described below. Facility-level teamwork and communication were stimulated to help institutionalise QI and develop a shared mental model that promotes high quality care standards among all staff.

#### Coordination between the BTSP and the Government of Bihar

It was in close coordination with the GoB that CARE India provided facility-level technical support and helped organise facility staff to use QI tools and strategies. Early in 2014, as the program transitioned from eight pilot districts to cover all 38 districts, CARE India teams were deployed to encourage and support ownership of the program by government counterparts through strategic engagement and techno-managerial assistance in operations planning and implementation. At the state level, a State RMNCHN+A Unit (SRU), established by CARE with representation from all development partners, constituted the technical back-up for the State Health Society of the Department of Health – the technical and executive body that manages virtually all GoB health programs. Operationally, regional, district, and block-level government health staff received orientation on RMNCHN epidemiology, life-saving interventions and their implementation solutions from CARE India’s district and regional field teams. Orientation sessions used participatory and experiential learning, and a variety of platforms such as field visits, skill stations, and classroom-based approaches. Strategic visioning exercises and cross-learning visits with government cadres to facilities in different districts were held to facilitate the development of block and district program implementation plans for the QI initiative.

Early in 2016, the size of the CARE India team expanded to include additional human resources primarily at the block and district levels – there was a manager per block and three officers at the district level (ie, a team lead, an officer addressing facility strengthening and another addressing outreach service strengthening), together called the District Resource Unit (DRU) and mirroring the SRU. Closely embedded within the government hierarchies, DRUs provided guidance to and closely supported the implementation of QI related policies and roll-out plans. A Strategic Program Management Team, composed of senior team leadership of the BTSP, SRU team leads and experienced program managers, supported and monitored the SRU and DRUs, focusing on progress towards meeting the strategic objectives of the program. Later, a web-portal was developed by the SRU for use by government functionaries, to streamline the supportive supervision process for the QI initiative, capture feedback from users, record service delivery problems and solutions in real-time, and stimulate discussions with government cadres. A separate measurement team called the Concurrent Measurement and Learning (CML) team collected data independent of the GoB and BTSP field implementation teams to provide time trends as well as explanatory deep dives into specific issues. Such continuous program monitoring provided a sense of formal data-driven management and coordinated decision-making to the initiative.

#### CARE India’s support to districts and facilities

CARE supported the activation of the previously defunct District Quality Assurance Committees (DQACs). Consisting of members with technical, managerial and financial functions, the 38 DQACs were authorised to oversee, monitor, and order changes to facilities within their district. An essential element of the QI initiative was the convening of facility-specific quality QI teams (QIT), which included facility managers, clinical and non-clinical staff. The QITs coordinated assessments, actions, and monitoring of the improvement process, using resources such as readiness toolkits and checklists with benchmarks. Before the implementation of the QI initiative, these different cadres rarely communicated or coordinated their activities. Soon after QITs were formed, they became part of an interdisciplinary team working with pride toward a shared goal of an improved facility. Based on annual assessments of facility readiness to provide EmONC services conducted by CARE India, gaps identified in human resources, supplies, infrastructure, and equipment were ranked and prioritised accordingly in facility action plans. QITs were functional in most (98%) public facilities across Bihar throughout 2014-2017, and were tasked with meeting urgent infrastructural needs, improving the workflow, implementing proposed interventions, meeting to review progress, addressing challenges, and setting new goals. QI coordinators from CARE India provided on-going guidance to QITs on how to best utilise available funds, make procurements, and unlock additional support from the GoB. Importantly, the QITs started working in synergy with the DQACs for infrastructure renovations and for procurement of drugs and equipment. At the end of 2017, DQACs in 28 of 38 (74%) districts in the state had conducted at least one meeting in the last 6 months to strengthen and sustain QI work in public facilities. An increasing number of facilities (58% in 2017 vs 42% in 2015) reporting being visited by DQAC members at least once per month (**Table 1**).

**Table 1:** Quality improvement strategies employed and corresponding indicators in Bihar, India

Category	Strategies	Indicators	Description +/− coverage			
**2015**	**2016**	**2017**	
**Management & coordination**	Coordination with GoB activities	Type of activities	Strategic visioning exercises and cross-learning visits by CARE with GoB cadres; prioritization of QI interventions	DRUs set up and embedded within GoB	Program web-portal to stimulate discussions with GoB cadres	
	DQACs and QITs established and functional	% facilities with QITs	~ 98%			
		Number of DQACs that met in the last 6 months	27 of 38 districts	29 of 38 districts		
		% facilities visited by DQAC members at least monthly	42%	48%	58%	
**Facility infrastructure**	Facility infrastructure improvements made	Type of improvements	“Cosmetic”; water and electricity upgrades; layout alteration to improve workflow	Blood banks improvements (licensure, infrastructure, staff training)	Sick newborn care units established in about half of the districts; 67 blood banks and 36 blood storage units functional	
**Contracting, procurement & inventory management capacity**	Processes to ensure availability of supplies and equipment streamlined	Type of processes	New procurement manual developed; technical specifications for equipment in place; registers for inventory management developed	E-Aushadhi rollout & training for users; linkages with BMSICL web-portal for real-time access to stock data	E-Aushadhi rollout continued; contract duration for supply purchases extended to 2 y; essential drug list updated; nationally accredited quality control laboratories established; review mechanism for BMSICL in place	
**Human resources**	→Staff gaps and mismatches identified and addressed	Type of activities	Rationalization of specialist doctors; nursing staff switched from contractual to permanent positions	New human resource policy designed; improved remuneration for doctors and nurses	Electronic statewide HRIS database designed; rationalization of positions prioritizing recruitment based on need; public health management cadres program designed	
**Clinical skills**	→Clinical capacity building of facility staff in BEmONC & CEmONC facilities through in-service training and mentoring	# BEmONC facilities where intervention has been completed	319 facilities (2385 ANMs & 308 Grade A nurses trained)			
		# trainers deployed in BEmONC facilities	10 master trainers & ~ 100 nurse-mentors			
		# CEmONC facilities where intervention has been completed	22 DHs & 1 medical college (277 nurse & 195 doctor mentees trained)			
		# trainers deployed in CEmONC facilities	5 master trainers & 4 doctor-mentors	6 master trainers & 6 doctor-mentors	5 master trainers & 5 doctor-mentors	
	→Clinical reviews of patients with complications	% facilities conducting monthly clinical reviews	n/a	>80%		
		average # days per month when clinical reviews done	2 d			
	→Capacity building for staff on FP	# facilities targeted	31 PHCs & RHs	~ 440 (60% of PHCs & 80% of RHs)		~ 527 (75% of PHCs & 85% of RHs)
		# providers trained in minilaparotomy/ tubal ligation	n/a	700		770
	→Real-time identification and tracking of “weak” newborns in the community	# districts targeted	Launched in 5 districts	All 38 districts		
**Data systems for decision-making**	Modernizing data systems	Type of health data systems	Statewide, centralized data dashboard (HSPT) designed; CARE’s CML functional; state- and district-level data dashboard with key indicators in use; standard patient registers and case sheet for patient triage across public facilities in use	District ranking and supervisory tools added to data dashboards		HMIS data improvements
	Continuous program monitoring	Type of data collection activities	CML household surveys; comprehensive facility assessments; AMANAT evaluation; special studies			

AMANAT – *Apatkalin Matritvaevam Navjat Tatparta* (translated Emergency Maternal and Neonatal Care Preparedness), ANM – auxiliary nurse-midwife, BMSICL – Bihar Medical Services and Infrastructure Corp. Ltd, BEmONC – basic emergency obstetric and newborn care, CEmONC – comprehensive emergency obstetric and newborn care, CML – Concurrent Monitoring and Learning, DH – district hospital, DQAC – District Quality Assurance Committee, DRU – District Resource Unit, GoB - Government of Bihar, HMIS – Health Management Information System; HRIS – Human Resource Information System, HSPT – Health Systems Progress Tracker, n/a – not available, QI – quality improvement, QIT – quality improvement team; PHC – primary health center, RH – referral hospital, TSU – Technical Support Unit

### Specific QI strategies and key results

#### Improving facility infrastructure

In addition to need-based improvements of the painting on walls to make the facilities more welcoming to patients and of water sources and electricity back-ups, facility layouts were analysed and altered to make them more functional in offering quality care and to better ensure patients’ privacy (**Table 1**). Installing elbow taps and digging pits for disposal of bio-medical waste helped improve infection control. Corrections were made by zoning operating room areas in higher than PHC-level facilities; meeting the requirements of each zone; developing sterile supply units; and streamlining the flow of patients, supplies, and staff. All these changes likely helped improve the morale of the staff as well.

Special Newborn Care Units with basic functionality were established in about half of the districts at the end of 2017. In coordination with the AIDS Control Society in Bihar, efforts were made in 2016 to strengthen the infrastructure and licensure process for blood banks. Standard operating procedures and registers were developed, and technical and logistical support were provided for training medical officers and laboratory technicians working in blood banks across the state. By the end of 2017, 67 blood banks (of which 28 were in the public sector) were operational, and an additional 36 blood storage units were approved to function in Bihar.

#### Building contracting, procurement and inventory management capacity

Persistent bottlenecks in the supply chain of critical commodities and equipment were identified, and the SRU initiated support to facilities for inventory and logistics management (**Table 1**). During 2015-2016, a technically competent BTSP team was embedded within the Bihar Medical Services and Infrastructure Corp. Ltd (BMSICL), which had handled equipment and supplies procurement in the state since 2010, to help streamline rules and processes. A comprehensive procurement manual was prepared to outline procurement procedures, technical specifications were developed for >200 types of equipment, and a team of consultants was assembled to bring in lessons from other states. Assistance was also offered in the design of registers and records for inventory management, and their use was mandated by the GoB in all public facilities.

**Figure 1:**
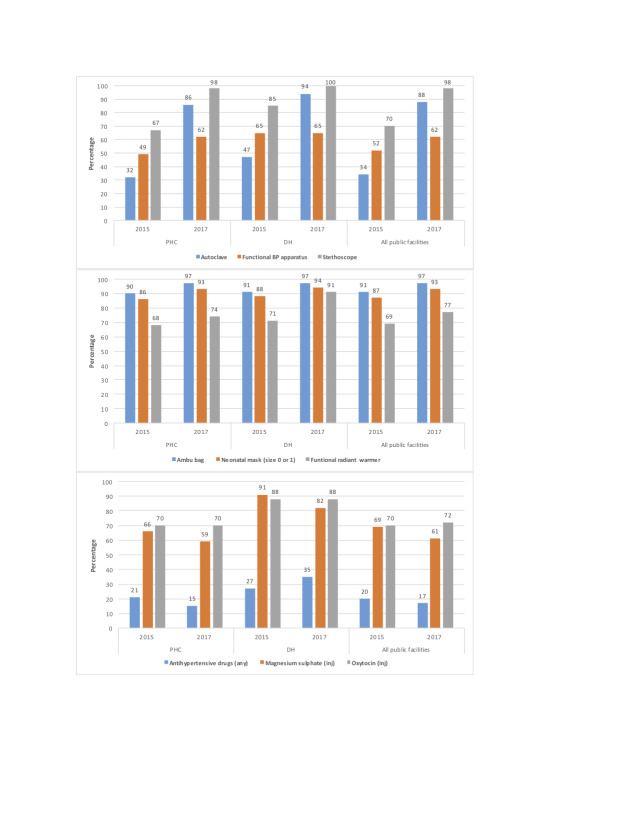
Changes in availability of equipment and supplies: Bihar, 2015 vs 2017. Data are from 2015 and 2017 Comprehensive Facility Assessments in public facilities. All round-to-round differences are statistically significant at *P* < 0.05. DH, district hospital; PHC, primary health center.

The BTSP supported the GoB and BMSICL in the rollout of e-Aushadhi, a statewide electronic inventory management system for medical supplies. In 2016, the BTSP trained master trainers who would eventually train all district and block-level users of this software as well as pharmacists in 10 districts; the GoB trained pharmacists in the other 28 districts with technical support from BTSP teams. Stock entries for district warehouses were completed, and by linking the e-Aushadhi to the BMSICL web-portal, stock information became accessible in real-time. Data from 2015 and 2017 cross-sectional Comprehensive Facility Assessments show that the availability of equipment (eg, autoclaves, blood pressure monitors, stethoscopes, radiant warmers), supplies (eg, ambu bags, neonatal masks) and drugs improved significantly over time (**Figure 1**). Higher-level facilities, such as district hospitals, fared better than PHCs. Supplies needed for key EmONC interventions (eg, uterotonics, magnesium sulfate) were less frequently out-of-stock than other supplies (eg, antihypertensives).

Through continued technical support to the BMSICL, in 2017, the BTSP helped finalise rate contracts for more than 90 drugs and standardised technical and financial qualification criteria for equipment purchases. BMSICL’s Board Committee approved an extension in duration of contracts for drug and equipment purchases to two years. To allow purchases from the Government of India’s centralised procurement portal (Government e-Marketplace), the BTSP facilitated state-level trainings for state and district health staff. The SRU team was also involved in updating the essential drug list and supported the BMSICL in empaneling nationally accredited quality control laboratories. A periodic review mechanism for BMSICL was recommended by the GoB and established with support from the BTSP.

#### Rationalising human resources

A 2014 assessment of Human Resources Information System (HRIS) data identified gaps and mismatches in available staff in public facilities (**Table 1**). The poor distribution of clinical staff led to efforts in 2015 to rationalise postings of specialist doctors (eg, obstetricians, anesthesiologists, pediatricians) to ensure their availability in all district hospitals; large numbers of nursing cadres were switched from contractual to permanent appointments, and redundant positions were eliminated. While awaiting a comprehensive human resources policy, piecemeal changes were made to improve doctors’ and nurses’ remuneration, ensuring higher pay for specialist than non-specialist doctors, facilitating their recruitment and retention in Bihar. Recruitment of additional specialist doctors on a contractual basis was needed to ensure their availability in facilities offering comprehensive EmONC services. To support an efficient use of human resources for health, in 2017, CARE India offered technical support in designing a more functional electronic HRIS database with state ownership.

**Figure 2:**
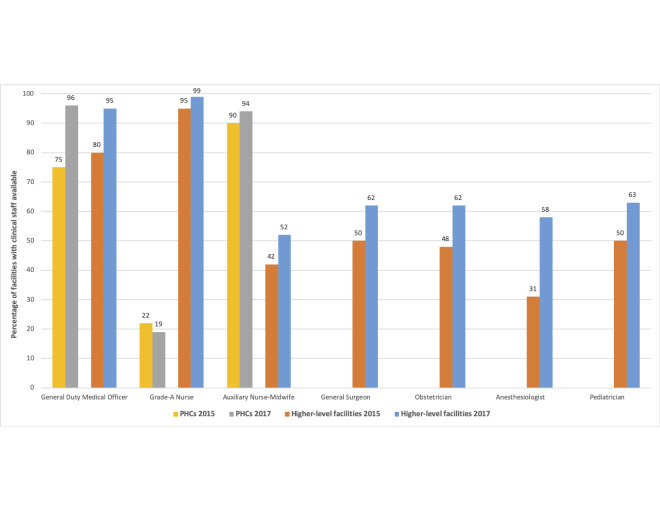
Availability of at least one clinical staff by type and facility level: Bihar, 2015 vs 2017. Data are from 2015 and 2017 Comprehensive Facility Assessments in public facilities. All round-to-round differences are statistically significant at *P* < 0.05. PHC, primary health center.

Data from the Comprehensive Facility Assessments documented that over 95% of functional public sector facilities in Bihar had at least one medical officer in 2017, in line with national requirements (**Figure 2**). Yet, despite progress, 80% of PHCs did not have grade-A nurses and about 40% of high-level facilities had unfilled specialist doctor positions that same year.

#### Improving providers’ clinical skills

Given the large number of nurses, especially auxiliary nurse-midwives, lacking adequate training and skills in offering EmONC services [5,6,15,16], there was great need to improve the skills and capacity of health workers. First, in all public facilities, efforts were made to better organise service provision and improve infection control (eg, handwashing, glove use, decontamination, disinfection, sterilisation, waste disposal), basic clinical care, and record keeping practices. The key component of the QI initiative was the AMANAT intervention, described in detail in this series [15], which aimed to improve providers’ skills and adherence to evidence-based EmONC and family planning practices without disturbing service delivery (**Table 1**). Briefly, it offered 40-50 days of in-service, on-site training of nurses by highly skilled pairs of nurse-mentors over a period of 7-8 months. In addition to bedside mentoring to monitor normal labour, conduct deliveries, and offer essential newborn care, trainings also addressed the early recognition and management of obstetric and neonatal complications. Simulation-based training and practice on mannequins were also employed during trainings in collaboration with Pronto International [15-17]. Of note, the nurse mentoring intervention was piloted in 80 public facilities in the initial eight pilot districts between 2012-2014 and showed significant and sustained improvements after one year in nurses’ knowledge and clinical skills post-intervention [5,6,18]. Training modules developed during the pilot were revised, and electronic libraries of training manuals and skill-building videos were developed by the end of 2014. AMANAT was then implemented in four phases covering about 80 public facilities each during 2015-2017 and trained almost 3000 nurses statewide. Doctors’ mentoring was also piloted in five of the eight initial pilot districts, primarily focused on CEmONC in district hospitals, with special efforts in strengthening obstetric, pediatric and anesthetic skills through use of master doctor-mentors, pediatric nurse-mentors, and operating room nurse-mentors. Given the limited number of specialist doctor-mentors available, the CEmONC mentoring component of AMANAT only trained about 500 nurses and doctors in 23 CEmONC facilities. Of note, the GoB formed a State Task Force to monitor AMANAT’s implementation progress and outcomes.

**Figure 3:**
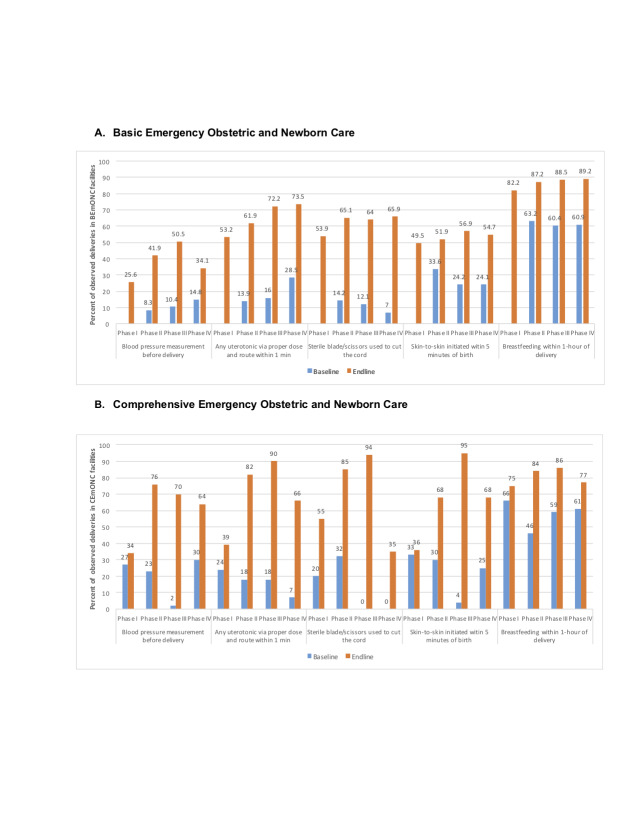
Changes in maternal and newborn practices before and after the AMANAT intervention: Bihar, 2014-2017. **Panel A.** Basic emergency obstetric and newborn care. **Panel B.** Comprehensive emergency obstetric and newborn care. Data are from Direct Observation of Deliveries conducted in conjunction with the four phases of the AMANAT intervention between 2014 and 2017. Phase I baseline data in Basic Emergency Obstetric and Newborn Care facilities were incomplete, thus not shown. All *P*-values comparing endline vs baseline levels are statistically significant at *P* < 0.05. AMANAT, *Apatkalin Matritvaevam Navjat Tatparta* (translated Emergency Maternal and Neonatal Care Preparedness).

To evaluate AMANAT, CARE India used provider knowledge assessments and direct observation of deliveries, the gold-standard for quality of care assessment [19]. Following AMANAT trainings, in both BEmONC and CEmONC public sector facilities, key indicators of intrapartum and essential newborn care improved substantially (**Figure 3**). While baseline levels were similar, post-intervention improvements in practices were greater in CEmONC than BEmONC facilities for intrapartum care, reaching similarly high levels (75%-89%) in both types of facilities for essential newborn practices like early initiation of breastfeeding.

Clinical reviews were initiated in most facilities exposed to the AMANAT intervention to facilitate provider communications and create opportunities for learning. The percentage of public facilities conducting clinical reviews on a monthly basis reached 80% in 2016 and 2017. An initiative for real-time identification, early management, family counseling, and tracking of “weak” (ie, birthweight <2000 g) newborns delivered in public hospitals in the community was piloted and launched in mid-2015 covering all public facilities except teaching hospitals. Daily phone calls to the families of these newborns and involvement of frontline health workers for home visits in the first week and beyond, if needed, were put in place. The initiative was fully absorbed into routine practice through GoB-issued guidelines in 2016, and included births at home, in public and private facilities.

Work to establish fixed days for family planning services in public facilities involved training providers to perform tubal ligation and vasectomy. These trainings took place in only 31 facilities in 2015, but involved 770 providers in 527 facilities in 2017. The intervention also aimed to streamline processes to ensure availability of supplies and equipment for these procedures.

#### Developing data systems for decision-making

In line with Government of India standards, the BTSP was instrumental in implementing use of standard client registers (eg, family planning, labour room, blood bank) across public facilities in Bihar. Work to improve the quality of data captured implied regular review of the data, feedback to staff completing the information, and adjustments based on discussions during unit reviews. A standardised patient case sheet that incorporates an algorithm for patient triage was widely implemented in 2015.

Since 2014 when CARE established the CML cell, it tracked implementation of various interventions to generate actionable program indicators, understand implementation barriers, and test solutions to these barriers [1,20]. A wide range of data have been collected regularly by CML (Table S1 in the **Online Supplementary Document**).

To overcome limitations with data availability and quality for the Health Management Information System (HMIS) [21], in 2015, CARE assisted the GoB to design a statewide, centralised data dashboard (ie, Health Systems Progress Tracker, HSPT; **Table 1**). It allows for multiple data sources to be searched and triangulated for data-driven decision-making. State- and district-level data dashboards were introduced to highlight key indicators and GoB cadres were trained to use them. District ranking was included in dashboards in 2016 to motivate district leadership to monitor program performance. A scoring system that assigned lesser or greater priority to different program indicators and incorporated relative indicator weights that can be modified to reflect program changes or new GoB priorities was used for ranking. Use of dashboard-integrated supervisory tools at block and sub-block levels allows for useful cross-linkages between GoB departments. For example, triangulation of data from CML household surveys and HMIS showed that there were 66 blocks with >50% home deliveries and that the practice of home deliveries, while spread across all blocks, typically occurred in pockets in villages or village clusters. A statewide initiative by the BTSP to identify home delivery pockets, understand, and address their causes led to a 6-percentage point increase in facility deliveries across the state between 2016 and 2017 (data not shown). Also of note, the BTSP assisted the GoB to improve identification and review of maternal deaths and their capture in HMIS. While only 600 deaths were reported during fiscal year 2015-2016, a total of 2049 and 1876 maternal deaths were identified in fiscal years 2016-17 and 2017-2018, respectively; of maternal deaths identified, 34% more were formally reviewed in 2017-2018 than in 2016-2017 (data not shown).

#### Changes in service utilisation in public vs private facilities

While not a program priority, utilisation of ANC has seen a sustained rise in Bihar since 2014, with virtually every pregnant woman reporting at least a check-up, 54% and 30% reporting 3+ and 4+ check-ups, respectively, in 2017 (Supplemental Figure 1 in the **Online Supplementary Document**). Improvements in ANC check-up content have also been documented. Two in three women had their blood pressure checked during pregnancy and >50% had an ultrasound examination in 2017, representing significant increases since 2014. Importantly, an increasing proportion of women who received clinical examination and tests during ANC did so in public facilities (Supplemental Figure 2 in the **Online Supplementary Document**). Overall, more blood pressure and weight measurements were taken during ANC in public than private facilities, while laboratory tests and ultrasound examinations were primarily done in the private sector.

**Figure 4:**
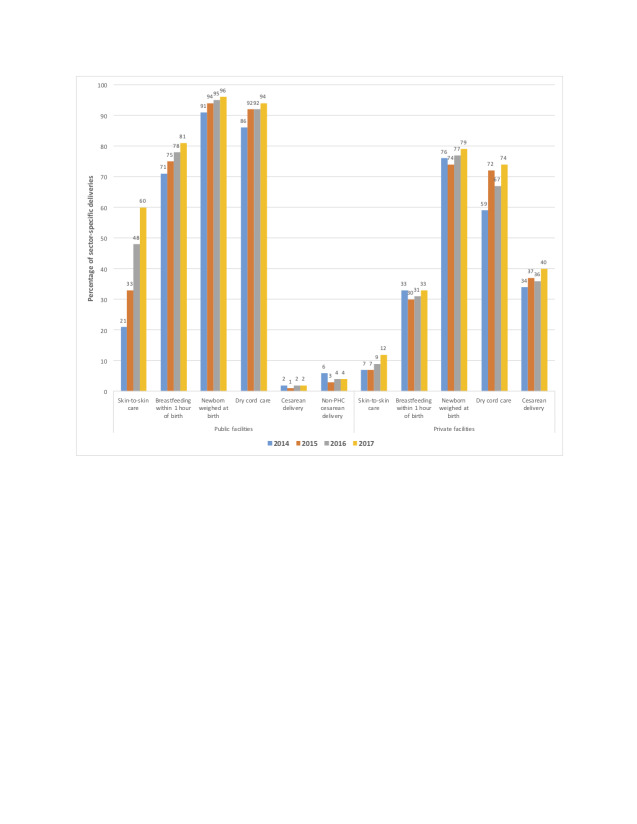
Changes in maternal and newborn health services in public vs private sector facilities: Bihar, 2014-2017. Data are from annual state-representative Community-based Household Surveys with women. Year-specific differences between public and private facilities are statistically significant at *P* < 0.05. PHC, primary health center.

During 2014-2017, 54%-59% of all deliveries in Bihar took place in public and 15%-17% in private sector facilities (Supplemental Figure 3 in the **Online Supplementary Document**). A 6-percentage point decline in home deliveries that occurred between 2016 and 2017 was accompanied by a higher increase in public than private facilities deliveries (5- vs 1-percentage points). Between 2014 and 2017, use of ambulances to reach public facilities for delivery increased only marginally from 13% to 17%, but the mean delay in receiving care upon reaching these facilities declined significantly from 21 to 14 minutes (data not shown). Most remarkably, essential newborn care practices such as skin-to-skin care, breastfeeding initiation <1 hour of birth, newborn weighing, and dry cord care were all more prevalent in public than private sector facilities during 2014-2017 (**Figure 4**). Yet, in 2017, only 2% of deliveries in PHCs and 4% in higher-level public facilities were by caesarean section compared to 40% in private facilities.

## DISCUSSION

The weak infrastructure, major and persistent staff shortages, and disruptions in procurement of equipment and supplies crippled the health system in Bihar for years. A large donor investment in sustained technical support in close coordination with GoB’s Health Department to implement a comprehensive, multi-dimensional QI initiative have improved the quality of RMNCHN in public facilities statewide in Bihar. The QI initiative drew heavily upon the work of QITs and, by and large, benefitted from input and regular visits by DQACs’ members. The initiative involved strengthening facilities’ infrastructure; developing or revising policies (eg, human resources, contracting and procurements); designing, implementing, and evaluating a training model that responds to immediate workforce capacity needs without disturbing service delivery (ie, AMANAT); and developing functional data systems (eg, e-Aushadhi, HRIS, HSPT, CML dashboards) to assist with program implementation, monitoring, and decision-making. Considerable efforts also went into building and maintaining GoB’s capacity and ownership of the program.

Overall, the results of this QI initiative are promising. A platform for continuous QI in RMNCHN has been established in public facilities, and work is on-going. An important and steady increase in institutional births until 2014 was followed by the 2014-2016 period with virtually no change in institutional births despite JSY program incentives [14,22], during which focus was on improving the quality of EmONC care in public facilities. In 2017, the BTSP finally targeted an improvement in institutional births – a decentralised, data-driven outreach strategy coupled with greater faith of the population in services offered in public facilities led to a significant increase in coverage of institutional deliveries in Bihar that year. The proportion of home deliveries declined in 2017; and of women delivering in health facilities, about 80% did so in the public sector. Based on facility assessments, the availability of critical supplies and functional equipment in these facilities improved over time. And, based on AMANAT evaluation and household survey data, the quality of RMNCHN services improved in public facilities, and for some services (eg, essential newborn care), it appears to be better than that offered in private facilities.

Nonetheless, considerable gaps still exist. While the high caesarean delivery rate in private sector facilities, much higher than recommended levels [23], is alarming, population-level estimates of caesarean rates are <10%. The capacity of public sector facilities to offer life-saving caesareans, currently at 2%-4%, needs to improve urgently and significantly. In line with findings from similar interventions in other Indian states [24-26], practice of evidence-based EmONC interventions improved substantially in facilities exposed to AMANAT trainings; moreover, there is evidence of sustained effects from AMANAT trainings in Bihar at one year [5,6]. Yet, documented improvements following this key QI intervention appear to be peaking at around 70% [15]. Proposed future AMANAT program strategies are being designed to address its sustainability.

Our assessment of the QI initiative in Bihar is not without limitations. Notably, an in-depth evaluation of the QI initiative and new data collection were beyond the scope of this study. However, we had access to and reviewed all available CARE India program documents and data and, in developing this article, benefitted from direct contributions and invaluable feedback from CARE India colleagues in Bihar who were directly involved with program planning and implementation. There is on-going debate in the scientific community regarding measurement of quality of care and best indicators to use for such measurement; thus, we used available relevant data for our assessment of the results of the QI intitiative. There were limitations in doing so. For example, to assess changes in available staff, equipment and supplies during 2014-2017, we used data from two statewide Comprehensive Facility Assessments conducted in 2015 and 2017. Because we did not have statewide information before 2014 when the initiative was initiated, we may be underestimating the true magnitude of changes before and after the QI initiative; also, with only two data points, it is difficult to ascertain the extent to which only temporary rather than consistent staff shortages or supply stockouts are being captured. The AMANAT intervention was implemented in a phased manner over the period of interest for this study, yet we pooled results from the various phases to examine changes in clinical practices before and after the intervention. Data from household surveys are self-reported by the women interviews, thus prone to information bias. Also of note, we did not aim to capture the variability in QI initiative results at district and block levels, which we know exists.

Implementation of this large statewide QI initiative in Bihar offers several lessons. The release of the national RMNCHN+A strategy and LaQshya guidelines considerably enhanced GoB’s interest and support for the QI initiative in Bihar. GoB’s ownership of the initiative, the coordination between the BTSP and GoB, and the technical support offered by the BTSP in each facility were key to the results observed. QI strategies and interventions selected for implementation built on each other and on the experience with the initial eight-district pilot phase. While the use of technical interventions helped mitigate the large knowledge gaps at all levels, understanding of the gaps in the “know-do” continuum improved over time, especially regarding AMANAT trainings, program monitoring, and data collection. BTSP’s strategy was to learn and adapt to the context rather than merely focus on the mechanics of QI implementation.

Challenges to implementing QI activities should be noted as well. Most importantly, the high turnover of clinical staff as well as GoB leadership posed significant challenges, but the self-driven, adaptive nature of the QI interventions implemented helped built a sense of ownership, responsibility, teamwork, and pride among all those involved. While the improvements in infrastructure were much needed and well received, malfunctioning equipment and supply stockouts still existed and had an impact on training interventions like AMANAT. Finding nurse-mentors and especially doctor-mentors for training was particularly difficult, and was a key reason for modifying the intervention early in 2018 to not rely on availability of mentors from outside the state. In addition, the demographic and geographic variability of the state required addressing contextual factors before QI activities were implemented.

Overall, our results are especially encouraging in light of only smaller QI initiatives previously conducted in other low- and middle-income countries having demonstrated modest success in improving some maternal and infant health outcomes [27-30]. In Ghana, “Project Fives Alive”' used a QI approach whereby process failures were identified by health staff and process changes were tested in the health facilities and corresponding communities to address those failures and to improve maternal and child health outcomes [27,28]. Among key results, improvements in early ANC and 4+ ANC visits for health posts as well as improvements in health education and patient triage in hospitals were associated with increased skilled delivery varying from 28% to 58% in these facilities’ catchment areas [27]. There was an association between early pregnancy identification and increased skilled delivery [28]. Also, improvements in postnatal care at health posts and health centres were associated with greater attendance of underweight infants at child welfare clinics [27]. In Tanzania and Uganda, a collaborative QI approach covering district, facility and community levels and supported by report cards generated through continuous household and health facility surveys was implemented to improve population-level coverage and quality of essential maternal and newborn services [29]. Using data from surveys conducted between 2011 and 2014, authors documented a 26% increase in the proportion of live births where mothers received uterotonics within 1 minute after birth in the intervention compared to the comparison district in Tanzania and an 8% increase in Uganda [29]. In addition, in Tanzania, the intervention was associated with a 31% increase in preparation of clean birth kits for home deliveries and a 14% increase in health facility supervision by district staff [29]. In Ethiopia, a quasi-experimental, QI health systems intervention in three pilot districts in Ethiopia identified, trained, and coached QI teams to develop and test change ideas to improve service delivery for mothers and newborns [30]. Preliminary results showed a significant positive increase in syphilis testing coverage after implementation of quality improvement changes for ANC, but this effect was not sustained over time [30]. Other positive impacts, albeit not statistically significant, were found from women’s attending 4+ ANC visits, having normal than low birthweight infants and initiation of kangaroo care [30]. Taken together, these early QI studies indicate promise, but also a need for adaptation, refinement and testing of specific strategies in local contexts.

## CONCLUSIONS

Substantial advances were made in improving the quality of RMNCHN services in public facilities in Bihar, and continued improvement that builds on the established QI platform is expected. Future QI work should aim to establish a culture of patient safety, sustained high quality, and respectful care in the state. New and innovative ways to deliver such care should be guided by data from now functional data systems and a deep understanding of demographic and geographic variability in Bihar.

## Additional material

Online Supplementary Document
