# Supplementary material for: Statewide implementation of a quality improvement initiative for reproductive, maternal, newborn and child health and nutritionin Bihar, India
Source: J Glob Health. 2020 Dec 19;10(2):021008. doi: 10.7189/jogh.10.021008 (PMC7759019; doi:10.7189/jogh.10.021008)
Supplement: Online Supplementary Document [file jogh-10-021008-s001.pdf]

Supplemental Table 1. Data sources used for assessing results of the quality improvement initiative in Bihar, India

Data source	Purpose	Frequency and representativeness	Methods of data collection	Data elements captured
**AMANAT -- Direct observation of deliveries[1]**	Explore providers' skills, practices, and quality of care offered in facilities	Pre- and post- AMANAT training in BEmONC and CEmONC facilities; 4 phases (319 facilities total) between 2015-2017; 36 of 38 districts in the state	Direct observation of deliveries by trained nurses serving as evaluators	Infection control measures, intrapartum and essential newborn clinical practices
**Comprehensive Facility Assessments[2]**	Identify gaps in health facilities' readiness for service delivery	Conducted by CARE's CML in all public facilities where deliveries are conducted; all 38 districts in the state; 3 rounds, about annual between 2015-2017	Direct observation of infrastructure (condition of wall, floor, roof, illumination), functionality of equipment, physical counting of drugs and consumables; review of records for completeness, up-to-date status and information related to case load, length of stay and bed occupancy	Infrastructure, human resources, availability and functionality of equipment, supplies (drugs and consumables), infection control, laboratory facility*, operation theatre*, referral system*
**Household Surveys[3]**	Assess women's and their children's receipt of care	Annually during 2014-2017; state-representative	Self-reported information by women with live infants 0-2 months old during household interviews	Health service utilization, counseling and advice received from frontline health workers

AMANAT - *Apatkalin Matritvaevam Navjat Tatparta* (translated Emergency Maternal and Neonatal Care Preparedness), BEmONC, basic emergency obstetric and newborn care, CEmONC - comprehensive emergency obstetric and newborn care, CML - Concurrent Monitoring and Learning, GoB - Government of Bihar, HMIS - Health Management Information System

[1]All data used in analyses presented.

[2]Data from 2015 and 2017 surveys used in analyses presented.

[3]Data from all 4 rounds conducted between 2014 and 2017 used in analyses presented.

*2017 data only.

Supplemental Figure 1. Changes in antenatal care attendance and content indicators: Bihar, 2014-2017

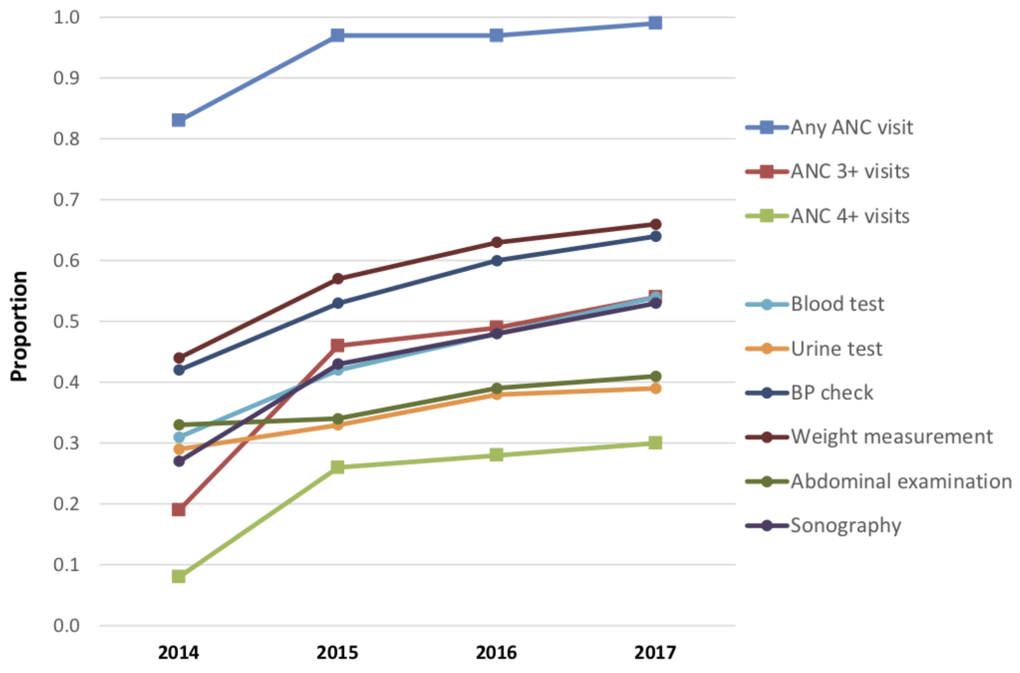

Notes: Data are from annual state-representative Community-based Household Surveys with women. For antenatal content, data are proportions of women with at least one ANC visit. All p-values for trend<0.05.

ANC, antenatal care; BP, blood pressure.

Supplemental Figure 2. Antenatal care content by source of care: Bihar, 2016 and 2017

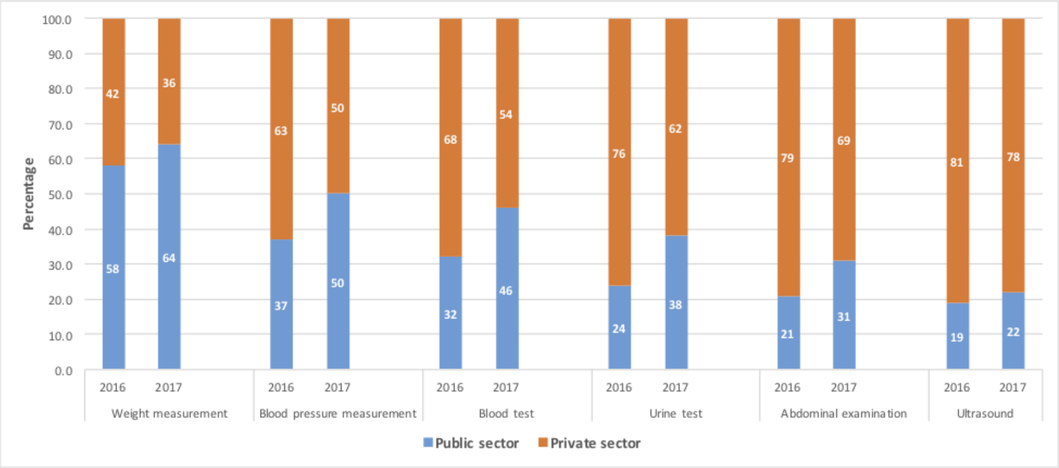

Notes: Data are from annual state-representative household surveys with women. For antenatal content, data are proportions of women with at least one antenatal care visit. Differences between 2016 and 2017 surveys are statistically significant at p-value<0.05.

Supplemental Figure 3. Changes in institutional deliveries and use of ambulances: Bihar, 2014-2017

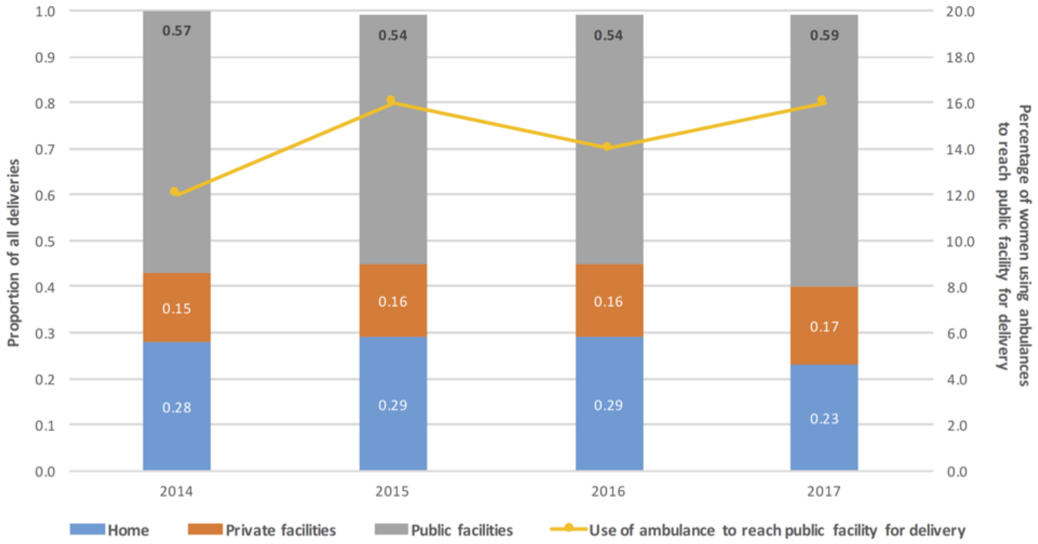

Notes: Data are from annual state-representative Community-based Household Surveys with women. Year-to-year differences in place of delivery are statistically significant at p-value<0.05.
